# Depression, Stressful Life Events, and the Impact of Variation in the Serotonin Transporter: Findings from the National Longitudinal Study of Adolescent to Adult Health (Add Health)

**DOI:** 10.1371/journal.pone.0148373

**Published:** 2016-03-03

**Authors:** Brett C. Haberstick, Jason D. Boardman, Brandon Wagner, Andrew Smolen, John K. Hewitt, Ley A. Killeya-Jones, Joyce Tabor, Carolyn T. Halpern, Beverly H. Brummett, Redford B. Williams, Ilene C. Siegler, Christian J. Hopfer, Kathleen Mullan Harris

**Affiliations:** 1 Institute for Behavioral Genetics, University of Colorado at Boulder, Boulder, Colorado, United States of America; 2 Institute of Behavioral Science, University of Colorado at Boulder, Boulder, Colorado, United States of America; 3 Department of Sociology, University of North Carolina, Chapel Hill, North Carolina, United States of America; 4 Carolina Population Center, University of North Carolina, Chapel Hill, North Carolina, United States of America; 5 Department of Maternal and Child Health, Gillings School of Global Public Health, University of North Carolina, Chapel Hill, North Carolina, United States of America; 6 Department of Psychiatry and Behavioral Sciences, Duke University Medical Center, Durham, North Carolina, United States of America; 7 Department of Psychiatry, Health Sciences Center, University of Colorado, Denver, Colorado, United States of America; University of Pennsylvania, UNITED STATES

## Abstract

**Background:**

The low transcriptionally efficient short-allele of the 5HTTLPR serotonin transporter polymorphism has been implicated to moderate the relationship between the experience of stressful life events (SLEs) and depression. Despite numerous attempts at replicating this observation, results remain inconclusive.

**Methods:**

We examined this relationship in young-adult Non-Hispanic white males and females between the ages of 22 and 26 (n = 4724) participating in the National Longitudinal Study of Adolescent to Adult Health (Add Health) with follow-up information every six years since 1995.

**Results:**

Linear and logistic regression models, corrected for multiple testing, indicated that carriers of one or more of the S-alleles were more sensitive to stress than those with two L-alleles and at a higher risk for depression. This relationship behaved in a dose-response manner such that the risk for depression was greatest among those who reported experiencing higher numbers of SLEs. In *post-hoc* analyses we were not able to replicate an interaction effect for suicide ideation but did find suggestive evidence that the effects of SLEs and 5HTTLPR on suicide ideation differed for males and females. There were no effects of childhood maltreatment.

**Discussion:**

Our results provide partial support for the original hypothesis that 5-HTTLPR genotype interacts with the experience of stressful life events in the etiology of depression during young adulthood. However, even with this large sample, and a carefully constructed a priori analysis plan, the results were still not definitive. For the purposes of replication, characterizing the 5HTTLPR in other large data sets with extensive environmental and depression measures is needed.

## Introduction

By the year 2030, depressive disorders are projected to rank first among disorders contributing to the global disease burden [1]. Major depression is a complex and heterogeneous disorder with a highly variable course. Lifetime prevalence rates vary significantly by age and sex, with young adults and females having higher rates of depression than males and older adults [2–4].

Stressful life events (SLE) are one of the most consistently documented exposures among those with depression [5–8]. Stressful events can include, but are not limited to, unemployment, acute or significant health problems, unexpected or persistent financial hardship, and relationship problems. While these are common experiences, not everyone who experiences them becomes depressed. Although the nature, kind, and timing of stressful life events have been implicated as sources of variability in the relationship between depression and SLEs, so too have biological factors such as serotonergic functioning [9, 10].

The neurotransmitter serotonin (5-HT) is synthesized in the brain by a small number of neuronal fibers with profuse projections throughout the brain, spinal cord and nervous system [10]. Because of this wide distribution, it has been implicated in a variety of mental and physical disorders [11–13]. Through removal of the 5-HT molecule at the synapse, the serotonin transporter (5-HTT) plays an important role in determining the extent and duration of serotonergic signaling. An insertion/deletion polymorphism (5HTTLPR) in the promoter region of the 5-HTT gene has been implicated as a source of variation in 5-HTT gene transcription rates such that the short (S) allele is less transcriptionally efficient than the long (L) allele [14, 15]. In 2003, Caspi et al [16] examined the role that 5HTTLPR polymorphism has in the relationship between SLEs and depression finding that carriers of one or more S-alleles were more likely to develop depression in the context of more SLE’s than those with two L-alleles.

Since that landmark study [16], there have been numerous replication attempts and three meta-analyses [17–24]. Differences in the conceptualization of stressful life events, depression, and their quantification have likely contributed to a literature that has not provided any definitive evidence for or against the original hypothesis. Small sample sizes, low statistical power, and an inconsistent preferred mode of examining the hypothesized interaction have also contributed to mixed findings [25], leaving some to contend that there have been few direct replication attempts of the original hypothesis [20, 21]. Here, we report findings from a study of the 5HTTLPR, stressful life events, and depression using an approach as close to an *a priori* direct replication as we could achieve in a large prospective nationally representative sample. Our first test examined the hypothesis that carriers of one or more S-alleles would be more sensitive to stress than individuals carrying no S-alleles. In a *post-hoc* manner and similar to Caspi et al [16], we examined the role of the 5HTTLPR in moderating the impact of stressful life events on suicide ideation and moderating the impact of childhood maltreatment on self-reported diagnosed depression. Based on prior published findings since Caspi et al [16] showing that the stress-moderating effects of the 5HTTLPR may differ between males and females [21, 26–31], we also examined sex as a moderator of stressful life events x 5HTTLPR effects on depression and suicidal ideation in *post-hoc* analyses.

## Methods

### Subjects

Add Health is a nationally representative, probability-based survey of adolescents in the United States, who were aged 12–19 years in the 1994–1995 school year, when the study began. A detailed description of the study design and the sampling strategy utilized in Add Health is available elsewhere [32,33]. At Wave III, 52.8% of the sample was male and the mean age was 22.0 (± 1.77, range 18–26) years old. The majority (67.1%) self-reported White race, 23% reported Black, and 9.9% either Asian or Native American. The participation rate was 77% [32]. Our analysis sample for the current study included only White males and females between 22 and 26 years of age participating in Wave III (2001–2002) and for whom we had genotype data to replicate Caspi et al [16] and avoid population stratification (n = 4724). Written informed consent was obtained at Wave III and protocols approved by the Institutional Review Board of the University of North Carolina at Chapel Hill. The Institutional Review Board at the University of Colorado Boulder approved the current study.

### Assessment

#### Stressful life events

Life events captured five domains of experiences: health, housing, employment, finance and relationships. Health-related events included hospitalization, violent injuries, frequency of emergency room visits, and being diagnosed with cancer. Housing-related events included residency changes (local, national), homelessness, shelter use, and whether the respondent had been evicted. Employment-related events included being unemployed, seeking job assistance, or receiving supplemental income. Finance-related events included being unable to pay for utilities, rent or mortgage; needing medical or dental help but being unable to pay for it, or having utility service cutoff. Relationship-related events included having experienced a violent relationship, divorce, or the breakup of a cohabitation living arrangement. A total of 21 items was used to create our stressful life events scale (SLE). For these five domains, 22.2% of the analysis sample reported having health related problems and 39.3% reported having employment related problems. A total of 34.4% reported having financial difficulties, 35.3% reported having housing problems, and 35.4% reported having problems in a significant relationship. S1 Table provides a comparison of the measured stressful life events in the current study and those by Caspi et al [16].

Following the original report by Caspi et al [16], if any item was endorsed within a domain, the respondent was given a score of 1 for that domain. Scores were then summed across all domains to produce a total number of stressful life events. Scores on this summed scale could range from 0 to 4.

#### Depression, suicide ideation, and childhood maltreatment

For our primary analysis, depression was assessed using responses from two questions. First, respondents were asked if they “*have been diagnosed with depression*.” Second, whether they “*have taken prescription medication for depression or stress*.” Responses were coded as yes or no (1/0) and yielded a categorical measure of depression. We felt this measure came closer to the clinical indicator of depression Caspi and colleagues [16] used. However, we also created a depressive symptom scale for *post-hoc* analysis using past-seven day depression symptoms, as assessed on the nine-item revised Center for Epidemiologic Studies Depression Scale (CES-D) [34]. Symptom endorsements were summed into a quantitative scale and ranged between 0 and 27.

Suicide ideation was assessed using two questions. First, “*during the past 12 months*, *had you ever seriously thought about committing suicide*” and “*during the past 12 months*, *how many times have you actually attempted suicide*.” Among respondents, 259 [5.5%] reported they had thought about committing suicide during the past year and 20.8% (n = 66) of those reported having attempted suicide. Rates of suicide ideation did not differ between males and females (χ2 (1) = 0.14, p = 0.71).

Maltreatment prior to the age of 12 was assessed using six questions. During the Wave III interview, participants were asked “*how often had your parents or other adult-caregivers left you home alone”*, *“how often had your parents or other adult care-givers not taken care of your basic needs”*, *“how often had your parent or other adult care-givers slapped*, *hit*, *or kicked you”*, *“how often had one of your parents or other adult care-givers touched you in a sexual way*, *forced you to touch him or her in a sexual way”*, *“how often had Social Services investigated how you were taken care of or tried to take you out of your living situation”*. Affirmative responses were coded as ‘1’ and ‘0’ otherwise. Responses were summed across all items and resulted in a scale that ranged from 0 to 6. Similar to Caspi et al (16) scores of 2 or more were collapsed and indicated ‘severe maltreatment.’

#### Genotyping

The 43 base-pair insertion/deletion polymorphism (5HTTLPR) in the 5’ regulatory region of the serotonin transporter gene (SLC6A4; 5HTT) and the A/G single nucleotide polymorphism (SNP) rs25331 in the long repeat unit of the 5HTTLPR was characterized from genomic DNA collected and isolated using the Oragene system (DNAgenotek, Kanata, Ontario, Canada) at Wave IV (2008–2009) in Add Health [35, 36]. Assay, allele and genotype information is provided in S1 Text.

#### Statistical analysis

Logistic regression models predicting depression and suicidality were as follows: *depression = b0 + b1(sex) + b2(5HTTLPR) + b3(SLE) + b4(5HTTLPRxSLE)*, where *b0* is the intercept, *b1* is the regression coefficient associated with the effect of sex (coded as 0 = male, 1 = female), *b2* is the regression coefficient associated with the influence of serotonin transporter genotype status (coded as 0 = s/s, 1 = s/l, 2 = l/l in order to reflect the number of “long” alleles), *b3* is the regression coefficient associated with the influence of stressful life events (SLE; coded as 0 = no SLEs, 1 = 1 SLE, 2 = 2 SLEs, 3 = 3 SLEs, 4 = 4+ SLEs), *b4* is the coefficient associated with the interaction effect and is the product of *5HTTLPR* genotype status and *SLE*. A p-value of 0.05 was used to determine statistical significance for *b4* in our primary analysis.

Analyses were run for two genotype classifications: *5HTTLPR* and *5HTTLPR* + *rs25531*. Because the study results did not differ between the two genotype classifications, we present findings from the analyses using the *5HTTLPR* + *rs25531* SNP classification (designated as: 5HTTLPR’). All analyses were conducted using the survey command in STATA (Version 12) and took into account the sampling design of Add Health. The independent (stressful life events, childhood maltreatment, 5HTTLPR genotype) and dependent (depression, suicide ideation) variables were developed independently, and the analyses were planned and reviewed by a panel of six investigators prior to testing in order to eliminate the possibility of ‘fishing expeditions’ in the data. For quantitative outcomes (CES-D depression symptomology scale), we used linear regression. Because our study was designed to test the gene-environment interaction hypothesis by Caspi et al [16], we corrected the statistical significance of all interaction analyses following our primary test using the conservative Bonferroni method. This resulted in a significance value for post-hoc tests of p = 0.005.

## Results

We examined the gene-environment hypothesis in a sample of white young adult males and females, ages 22–26, who participated in Wave III (2001–2002) of Add Health (n = 4724). In our analysis sample, 23.6% (n = 1117) had two copies of the short allele (s/s genotype), 48.8% (n = 2303) had one copy of the short allele (s/l genotype), and 27.6% (n = 1304) had no copies of the short allele (l/l genotype). The three-genotype groups were in Hardy-Weinberg equilibrium (χ2 = 2.597, df = 1, p = 0.11) and there were no significant differences in genotype frequencies between males and females (χ2 = 0.32, p = 0.85).

Stressful life events were assessed using 21 separate events including health, housing, employment, financial, and relationship experiences. Seventy-nine percent of our sample reported experiencing at least 1 stressful life event and of those, 23.3% reported 2 events, 12.7% reported 3 events, and 7.2% experienced 4 or more. There were no significant differences in SLE scores (F(2,4723) = 0.41, p = 0.66) as a function of genotype status and indicated that the 5HTTLPR polymorphism was not associated with exposure to stressful life events.

Thirteen percent (n = 626) of our analysis sample self-reported being diagnosed with depression between 22 and 26 years of age. Females were more likely than males to report having a diagnosis of depression (odd ratio = 2.17; 95% confidence interval, (CI): 1.85 to 2.55) and report having taken medication for depression (odds ratio = 3.4, 95% CI: 2.36 to 3.91). Males and females were equally likely to report having spent time in a hospital for mental illness in the previous five years (odds ratio = 1.10, 95% CI: 0.74 to 1.63) and to have attempted or seriously thought about committing suicide in the past year (odds ratio = 0.70, 95% CI: 0.48 to 1.02).

**Fig 1:**
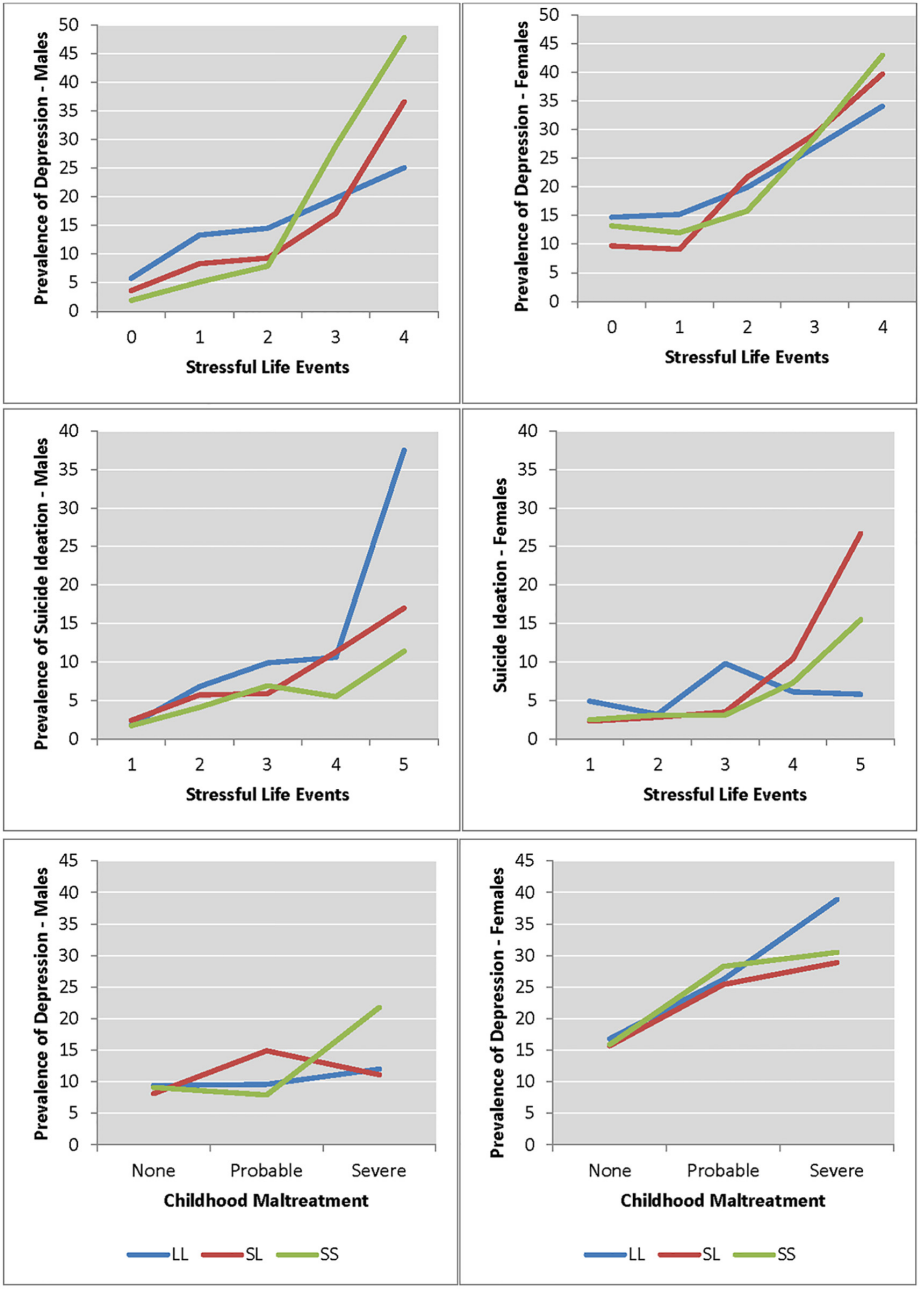
Prevalence Rates for Depression as a function of 5-HTTLPR’ genotype status, two stressful environmental experiences and suicide ideation among young adult males and females. **Figs A and B (Top Row)** graphically illustrate the prevalence of depression as a function of stressful life events; **Figs C and D (Middle Row)** illustrate the prevalence depression as a function of suicide ideation; and **Figs E and F (Bottom Row)** illustrate the prevalence of depression as a function of childhood maltreatment experiences.

We began our logistic regression analyses by examining whether the risk for depression increased as a function of 5HTTLPR genotype and the number of stressful life events experienced. Sample sizes for our primary and *post-hoc* analyses are provided in S2 Table. For both males and females, as the number of life events increased the risk for depression also increased. Prevalence estimates for depression were similar across 5HTTLPR genotype status, though depression risk appeared to increase more steeply for males who have one or more of the short alleles (Fig 1a and 1b, S3 Table). We interpreted this pattern of results as providing preliminary support for the originally hypothesized gene-environment interaction [16]. Formal tests of this possibility, accounting for the sampling design of Add Health, revealed a main effect for SLEs [b = 0.67, t = 8.75, p < 0.01 95% CI: 0.52 to 0.83], a main effect of the 5HTTLPR genotype [b = 0.40, t = -2.66, p < 0.01, 95% CI: 0.10 to 0.69], and for a statistically significant interaction [b = -0.16, t = 0.07, p = 0.02, 95% CI: -0.29 to -0.02] (Table 1). Post-hoc analyses of this replication finding revealed that while stressful life events were a risk factor for depression for both sexes, the role of the 5HTTLPR and its interaction with stressful life events approached significance (p = 0.007, 95% CI: -0.54 to -0.09) only for males [Fig 1a and 1b, Table 1]. A formal test of whether the interaction terms differed between the sexes revealed a non-significant sex-5HTTLPR-stressful life events interaction [b = 0.25, t = -1.82, p = 0.07, 95% CI: -0.02 to 0.53].

**Table 1:** Unstandardized Logistic Regression Estimates and Standard Errors Predicting Depression and Suicide Ideation.

	Depression Analysis for Full Sample				Maltreatment Analysis for Full Sample				Suicide Ideation Analysis for Full Sample			
	*b*		S.E.		*b*		S.E.		*b*		S.E.	
Intercept	-3.39		0.19†		-2.40		0.14†		-3.92		0.36†	
Sex (Female)	0.55		0.10†		0.70		0.11†		-0.27		0.16	
5HTTLPR’	0.40		0.15‡		0.08		0.07		0.32		0.23	
SLE	0.67		0.08‡						0.59		0.12†	
5HTTLPR’xSLE	-0.16		0.07§						-0.07		0.09	
Maltreatment					0.45		0.14‡					
5HTTLPR’x Maltreatment					-0.01		0.11					
	Depression Analysis				Maltreatment Analysis				Suicide Ideation Analysis			
	Males		Females		Males		Females		Males		Females	
	*b*	S.E.	*b*	S.E.	*b*	S.E.	*b*	S.E.	*b*	S.E.	*b*	S.E.
Intercept	-4.06	0.35†	-2.47	0.24†	-2.47	0.20†	-1.65	0.11†	-3.59	0.33†	-4.52	0.50†
5HTTLPR’	0.82	0.27‡	0.16	0.18	0.20	0.15	0.01	0.09	0.50	0.24	0.05	0.35
SLE	0.96	0.15†	0.52	0.08†					0.36	0.13‡	0.36	0.17†
5HTTLPR’xSLE	-0.33	0.12‡	-0.07	0.08					0.13	0.11	0.13	0.12
Maltreatment					0.43	0.24	0.48	0.15‡				
5HTTLPR’x Maltreatment					-0.10	0.22	0.03	0.13				

Note: 5HTTLPR’ genotype = 5HTTLPR + rs25531 snp; S.E., Standard Error. Uncorrected p-values are reported for post-hoc tests and did not remain significant following multiple test correction. Full regression results and 95% confidence intervals are provided for each analysis in S6 Table.

^†^ Significant at P < 0.001.

^‡^ Significant at P < 0.01.

^§^ Significant at P < 0.05.

### Post-hoc analyses

Following on the primary hypothesis that the 5HTTLPR moderated the relationship between stressful life events and depression, Caspi et al [16] investigated similar interaction hypotheses for depression related dependent and independent variables (suicide ideation and childhood maltreatment, respectively). In our sample of males and females, prevalence rates of suicidal ideation were highest among those who had experienced greater numbers of stressful life events (S4 Table) regardless of sex. Across 5HTTLPR genotype, however, the effect of stressful life events differed as a function of sex. For females carrying one or more of the S-alleles, prevalence of suicide ideation increased with an increasing number of stressful life events whereas for males, the prevalence rates increased for carriers of one or more L-alleles (S4 Table). For both sexes, neither 5HTTLPR genotype, nor its interaction with stressful life events was significantly associated with suicidal ideation (Table 1, Fig 1c and 1d), although this relationship approached significance for females (S5 Table). A formal test of whether the interaction terms differed between the sexes revealed a non-significant sex-5HTTLPR-stressful life events interaction [b = -0.33, t = 2.33, p = 0.02, 95% CI: -0.63 to -0.05] after multiple test correction. Similarly, a test of the differential effect of the 5HTTLPR as a function of sex that also controlled for depression status [b = -0.39, t = 2.78, p = 0.007, 95% CI: -0.67 to -0.11] was not significant after correcting for multiple testing. For childhood maltreatment, neither the 5HTTLPR genotype nor its interaction with maltreatment significantly predicted depression in this young adult sample (Table 1, Fig 1e and 1f, S6 Table).

Finally, we examined a quantitative depression score based on CES-D symptom counts (z-scored). Although stressful life events predicted CES-D depression symptoms [b = 0.18, SE = 0.05, t = 3.53, p = 0.001, 95% CI: 008 to 0.28], neither 5HTTLPR genotype [b = -0.01, SE = 0.06, t = 0.22, p = 0.82, 95% CI: -0.12 to 0.10] nor its interaction with SLEs [b = 0.03, SE = 0.05, t = 0.08, p = 0.94, 95% CI: -0.84 to 0.09] was significant.

## Discussion

In the current report we examined the hypothesis that 5HTTLPR genotype moderated the relationship between experiences of stressful life events and depression during young adulthood. We sought to test this hypothesis by adopting a direct replication strategy that included an analytic approach and a stressful life events measure similar to those employed previously [16]. Importantly, as a group we specified the exact tests that would be conducted prior to undertaking our analyses to limit the number of tests conducted and preserve the integrity of interpretation for those that were conducted. Results supported the hypothesis that the 5HTTLPR plays a role in moderating the impact of SLEs on depression status, though at a statistically significant level only in males and using a self-reported ‘ever’ diagnosis of depression. We did not find support for a similar role of the 5HTTLPR in the relationship between stressful life events and suicide ideation; however, post-hoc analyses were suggestive of a stress-moderating effect of the 5HTTLPR on suicide ideation that differed between males and females. Further, while having experienced maltreatment as a child was a significant risk for depression, we did not detect a significant maltreatment-5HTTLPR interaction in these data as had been reported previously [16]. Post-hoc considerations revealed, that in the context of increasing numbers of stressful life events, common variation in 5HTTLPR genotype could contribute to differences in mental health problems between the sexes.

In the absence of stressful life events, we found rates of depression that were similar across genotype status. This indicated that at low levels of stress, 5HTTLPR genotype was not related to the risk for depression. As the number of stressful life events increased, the prevalence of depression also increased among those carrying one or more copies of the short allele. This relationship between stressful life events and depression was most evident at the extremes of the distribution where sample sizes were smaller, though still larger than the original study [16]. Moreover, the higher prevalence of depression among those with many stressful life events was significant only for males. Finally, as new genetic variation in the 5HTTLPR had been identified since the original study [16] we included SNP rs25531 in our analyses. Doing so negligibly changed the observed relationships as compared with results including just the 5HTTLPR. This is consistent for a SNP with a low minor allele frequency (MAF) among Caucasians [> 5%; 35] such that the number of participants reclassified on the basis of functional binning of the 5HTTLPR was limited. This, however, would not be the expectation in genetically diverse samples with greater MAFs.

Gene-environment interaction studies offer an opportunity to understand biological mechanisms important for predicting risk and evaluating changes in modifiable environments [37–39]. Further, these kinds of studies can help to identify environmental effects that act primarily in genetically susceptible individuals. There have been many attempts at replicating the observation that the 5HTTLPR S-allele moderates the relationship between depression and stressful life events [16–20, 25], often with conflicting results. Similarly, findings from meta-analyses [20–22] have also been inconclusive, with some reporting no effect and others full support for the original hypothesis. Against this literature, our results agree with the notion that the interaction effect of 5HTTLPR genotype and stressful life events on depression is marginal and that the effect size is smaller than that originally reported. Further, similar to one prior study [19], our results suggest that it is important to capture the specific forms of life stress originally set forth in Caspi and colleagues [16] original report [40, 41].

Suicide ideation is often reported among patients with depression and those experiencing acute stress [42]. Because of the magnitude of impact suicide has on society there is a great interest in understanding its biological and social causes. To this end, our results suggest the possibility that in the presence of an increasing number of stressful life events different 5HTTLPR alleles may confer a susceptibility to suicide ideation that differs between males and females. If true, this finding would extend Caspi et al [16] and support previous studies showing sex moderation of the 5HTTLPR effects on depression [28, 30, 31] and negative affect [43], and may offer one explanation for the heterogeneity in association tests of the 5HTTLPR and suicide behaviors that implicate both the L-allele and S-allele [44, 45]. If replicated, these results would aid efforts to better identify those at risk for suicide and implicate environments and social experiences that may be modified to reduce such risk.

Similar to other replication attempts, our study is not without limitations. First, we were unable to establish a temporal relationship between the experience of a stressful life event and the onset or occurrence of depression. As such, some of the measured stressful life events may have occurred after the onset of depression, which may have the effect of biasing our interaction parameter [46]. Similarly, stressful life events examined here were retrospectively reported as having occurred sometime within the preceding five-years while depression diagnosis could have occurred at any point before the Wave III interview. Stressors most salient to depression are those which are acute, major, occurred within the previous six-months, and are participant focused [40, 41]. Although our stressful life events measure was very similar to that specified by Caspi and colleagues (16), we were not able to include each of its aspects. Second, our coding of SLEs, though exactly the same as Caspi et al [16], limits the potential importance of experiencing multiple types of SLE’s within one domain or multiple occurrences of the same event within a domain. This has, for example, the consequence of scoring someone who has experienced multiple forms of violence over years in an intimate relationship but no other SLE the same as a person who moved states for a job and no other SLE’s. Said differently, scoring SLE domains as we have limits the impact of the severity of an experience. A coding scheme that accounts for this by possibly weighting subsequent experiences may provide a better allocation of subjects to SLE groups and reduce sample heterogeneity among those experiencing fewer SLE’s. Third, though we were able to detect a significant interaction between the 5HTTLPR and stressful life events in a much larger sample than examined by Caspi and colleagues [16], the effect size was less robust. One potential reason for this could be differences between interview and self-reported adversity. Previous meta-analyses [45] and systematic reviews [39] have suggested that the use of interviews and/or multiple informants may afford better sensitivity for the detection of gene-environment (GxE) interactions. Fourth, reports of childhood maltreatment were retrospective and therefore may have been influenced by distorted memories or recall bias [47–50]. Lastly, for replication purposes [16] we restricted our sample to include only Caucasians and as such are unable to establish the extent to which findings would be similar in other race/ethnic populations. Given race differences in the prevalence of depression [4, 50] and allele frequencies of the functional SNP rs25531 [51, 52], examining this hypothesis in diverse ethnic samples should be a priority for future research.

In conclusion, by committee, we set out to examine the hypothesis that SLEs moderated the impact of the 5HTTLPR S-allele on depression among young adults. To do so, we attempted to approximate as closely as possible the measures originally examined by Caspi et al [16] in a large well-characterized sample followed longitudinally since adolescence. Towards this end, a notable advantage of the Add Health sample is the extensive environmental measures combined with the unique availability of simple sequence data [34]; in particular rs25531 and the 5HTTLPR. Although these types of samples provide strong statistical power for hypothesis testing, they may not always assess each phenotype of interest in as an extensive of a manner as smaller samples designed to test circumscribed hypotheses. As noted above, we could not approximate every phenotype as originally [16] put forward. Despite this, our results utilizing a highly similar measure of SLEs, a clinically orientated depression measure, a more transcriptionally accurate genotype classification, and the same analytic approach, we obtained evidence, albeit not unequivocal, that supported the original finding reported by Caspi et al [16]. Specially, young adults with lower *5HTT* transcription and greater numbers of SLEs reported higher rates of depression as compared with those carrying the L-allele and experiencing fewer SLEs. Although larger sample sizes are desirable to further explore the questions asked here, it is important to do so in samples with extensive environmental characterization using alternative methodologies and strategies that elucidate the connections between sequence variation, gene regulation, and depression related neural pathophysiology. In this way we can realize the identification of environmental effects that act in susceptible individuals.

## Supporting Information

S1 Table Domains and items for Stressful Life Events (SLE) Measure. (DOCX)

S2 Table Sample size and frequencies for independent and dependent variables—primary and *post hoc* analyses. (DOCX)

S3 Table Prevalence of depression as a function of 5HTTLPR genotype and number of stressful life events among Males and Females. (DOCX)

S4 Table Prevalence of suicide ideation as a function of 5HTTLPR genotype and number of stressful life events among Males and Females. (DOCX)

S5 Table Regression output for depression, childhood maltreatment, and suicide ideation analyses in the Full sample and among Males and Females. (DOCX)

S6 Table Prevalence of depression as a function of 5HTTLPR genotype and number of childhood maltreatment events among Males and Females. (DOCX)

S1 Text Genotyping information. (DOCX)

## References

[1] Matthers CD, Loncar D. [2006] Projections of global mortality and burden of disease from 2002 to 2030. *PloS Medicine* 3[11]: e442 17132052 10.1371/journal.pmed.0030442 PMC1664601

[2] Scott J, Dickey B. [2003] Global burden of depression: the intersection of culture and medicine. *British Journal of Psychiatry* 183: 93–94. 10.1192/bjp.183.2.92 12893658

[3] Adkins DE, Wang V, Dupre ME, van den Oord EJCD, Elder GH. [2009] Structure and stress: trajectories of depressive symptoms across adolescence and young adulthood. *Social Forces* 88: 31–60. 10.1353/sof.0.0238 PMC2812933 20119501

[4] Gonzalez HM, Tarraf W, Whitfield KE, Vega WA. [2010] The epidemiology of major depression and ethnicity in the United States. *Journal of Psychiatric Research* 44: 1043–1051. 10.1016/j.jpsychires.2010.03.017 20537350 PMC2963677

[5] Holmes TH, Rahe RH. [1967] The social readjustment rating scale. *J Psychosom Res* 11: 213–218. 6059863 10.1016/0022-3999(67)90010-4

[6] Kendler KS, Kessler RC, Walters EE, MacLean C, Neale MC, Health AC, et al [1995] Stressful life events, genetic liability, and onset of an episode of major depression in women. *Am J Psychiatry* 152: 833–842. 7755111 10.1176/ajp.152.6.833

[7] Kendler KS, Karkowski LM, Prescott CA. [1998] Stressful life events and major depression: risk period, long-term contextual threat, and diagnostic specificity. *J Nerv Ment Dis* 186: 661–669. 9824167 10.1097/00005053-199811000-00001

[8] Kessler RC. [1997] The effects of stressful life events on depression. *Ann Rev Psychol* 48:191–214. 9046559 10.1146/annurev.psych.48.1.191

[9] Caspi A, Hariri AR, Holmes A, Uher R, Moffitt TE. [2010] Genetic sensitivity to the environment: The case of the serotonin transporter gene and its implications for studying complex diseases and traits. *American Journal of Psychiatry*. 167: 509–527. 10.1176/appi.ajp.2010.09101452 20231323 PMC2943341

[10] Deneris E, Wyler SC. [2012] Serotonergic transcriptional networks and potential importance to mental health. *Nature Neuroscience* 15: 519–527. 10.1038/nn.3039 22366757 PMC3594782

[11] Jacobs N, Kenis G, Peeters F, Derom C, Vlietinck R, van Os J. [2006] Stress-related negative affectivity and genetically altered serotonin transporter function. *Arch Gen Psychiatry* 63: 989–996. 16953001 10.1001/archpsyc.63.9.989

[12] Wray NR, James MR, Gordon SD, Dumenil T, Ryan L, Coventry WL, et al [2009] Accurate, large-scale genotyping of 5HTTLPR and flanking single nucleotide polymorphisms in an association study of depression, anxiety, and personality measures. *Biol Psychiatry* 66: 468–476. 10.1016/j.biopsych.2009.04.030 19541292 PMC3060567

[13] Atmaca M, Onalan E, Yildirim H, Uyce H, Koc M, Korkmaz S, et al [2011] Serotonin transporter gene polymorphism implicates reduced orbito-frontal cortex in obsessive-compulsive disorder. *J Anxiety Disord* 25: 680–685. 21441009 10.1016/j.janxdis.2011.03.002

[14] Heils A, Teufel A, Petri S, Stober G, Riederer, Bengel D, Lesch KP [1996] Allelic variation of human serotonin transporter gene expression. *J Neurochem* 66: 2621–2624. 8632190 10.1046/j.1471-4159.1996.66062621.x

[15] Lesch KP, Bengel D, Heils A, Sabol SZ, Greeberg BD, Petri S, et al [1996] Association of anxiety-related traits with a polymorphism in the serotonin transporter gene regulatory region. *Science* 274: 1527–1531. 8929413 10.1126/science.274.5292.1527

[16] Caspi A, Sugden K, Moffitt TE, Taylor A, Craig IW, Harrington H, et al [2003] Influence of life stress on depression: moderation by a polymorphism in the 5-HTT gene. *Science* 301: 386–389. 12869766 10.1126/science.1083968

[17] Coventry WL, James MR, Eaves LJ, Gordon SD, Gillespie NA, Ryan L, Heath AC, et al [2009] Do 5HTTLPR and stress interact in risk for depression and suicidality? Item response analyses of a large sample. *Am J Med Genet Part B*. 153B: 757–765. 10.1002/ajmg.b.31044 PMC3319106 19911410

[18] Gillespie NA, Whitfield JB, Williams B, Heath AC, Martin NG. [2005] The relationship between stressful life events, the serotonin transporter (5-HTTLPR) genotype and major depression. *Psychol Med* 35: 101–111. 15842033 10.1017/s0033291704002727

[19] Kendler KS, Kuhn JW, Vittum J, Prescott CA, Riley B. [2005] The interaction of stressful life events and a serotonin transporter polymorphism in the prediction of episodes of major depression. *Arch Gen Psychiatry*. 62: 529–535. 15867106 10.1001/archpsyc.62.5.529

[20] Munafo MR, Durrant C, Lewis G, Flint J. [2009] Gene x environment interactions at the serotonin transporter locus. *Biol Psychiatry* 65: 211–219. 10.1016/j.biopsych.2008.06.009 18691701

[21] Risch N, Herrell R, Lehner T, Liang KY, Eaves L, Hoh J, et al [2009] Interaction between the serotonin transporter gene (5-HTTLPR), stressful life events, and risk of depression. *JAMA*. 301[23]: 2462–2471. 10.1001/jama.2009.878 19531786 PMC2938776

[22] Karg K, Burmeister M, Shedden K, Sen S. [2011] The serotonin transporter promoter variant (5-HTTLPR), stress, and depression meta-analysis revisited. *Arch Gen Psychiatry* 68: 444–454. 10.1001/archgenpsychiatry.2010.189 21199959 PMC3740203

[23] Uher R, McGuffin P. [2010] The moderation by the serotonin transporter gene of environmental adversity in the etiology of depression: 2009 update. *Mol Psychiatry* 15: 18–22. 10.1038/mp.2009.123 20029411

[24] Beaver KM, Vaughn MG, Wright JP, Delisi M. [2012] An interaction between perceived stress and 5HTTLPR genotype in the prediction of stable depressive symptomatology. *Am J Orthopsychiatry* 82[2]: 260–266. 22506528 10.1111/j.1939-0025.2012.01148.x

[25] Duncan LE, Keller MC. [2011] A critical review of the first 10 years of candidate gene-by-environment interaction research in psychiatry. *Am J Psychiatry* 168: 1041–1049. 10.1176/appi.ajp.2011.11020191 21890791 PMC3222234

[26] Ely TC, Sugden K, Corsico A, Gregory AM, Sham P, McGuffin P, Plomin R. et al [2004] Gene-environment interaction analysis of serotonin system markers with adolescent depression. *Mol Psychiatry* 9: 908–915. 15241435 10.1038/sj.mp.4001546

[27] Chorbov VM, Lobos EA, Todorov AA, Health AC, Botteron KN, Todd RD. [2007] Relationship of 5-HTTLPR genotypes and depression risk in the presence of trauma in a female twin sample. *Am J Med Genet* 144B: 830–833. 17455215 10.1002/ajmg.b.30534

[28] Sjoberg RL, Nilsson KW, Nordquist N, Ohrvik J, Leppert J, Lindstrom L, Oreland L. [2006] Development of depression: sex and the interaction between environment and a promoter polymorphism of the serotonin transporter gene. *Int J Neuropsychopharamcol* 9: 443–449. 10.1017/S1461145705005936 16212676

[29] Grabe HJ, Lange M, Wolff B, Volzke H, Lucht M, Freyberger HJ, et al [2005] Mental and physical distress is modulated by a polymorphism in the 5-HT transporter gene interating with social stressors and chronic disease burden. *Mol Psychiatry* 10: 220–224. 15263905 10.1038/sj.mp.4001555

[30] Brummett BH, Boyle SH, Siegler IC, Kuhn CM, Ashley-Koch A, Jonassaint CR, et al [2008] Effects of environmental stress and gender on associations among symptoms of depression and the serotonin transporter gene linked polymorphism (5-HTTLPR). *Behav Genet* 38: 34–43. 17955359 10.1007/s10519-007-9172-1 PMC2777886

[31] Gonda X, Juhasz G, Laszik A, Rihmer Z, Bagdy G. [2005] Subthreshold depression is linked to the functional polymorphism of the 5HT transporter gene. *J Affect Disord* 87: 291–297. 16002148 10.1016/j.jad.2005.05.007

[32] Harris KM, Halpern CT, Smolen A, Haberstick BC. [2006] The National Longitudinal Study of Adolescent Health (Add Health) twin data. *Twin Res Hum Genet*, 9: 988–997. 17254442 10.1375/183242706779462787

[33] Harris KM. Design features of Add Health [2011]. http:www.cpc.unc.edu/projects/addhealth/data/guides/design%20paper%20WI-IV.pdf

[34] Radloff L. [1977] The CES-D Scale: a self-report depression scale for research in the general population. *Appl Psychol Meas* 385–401.

[35] Haberstick BC, Smolen A, Stetler GL, Tabor JW, Roy T, Casey HR, Pardo A, Roy F, Ryals LA, Hewitt C, Whitsel EA, Halpern CT, Killeya-Jones LA, Lessem JM, Hewitt JK, Harris KM. [2014] Simple sequence repeats in the national longitudinal study of adolescent health: an ethnically diverse resource for genetic analyses of health and behavior. *Behav Genet* 44: 487–497. 10.1007/s10519-014-9662-x 24890516 PMC4244076

[36] Haberstick BC, Smolen A, Williams RB, Bishop GD, Foshee VA, Thornberry TP, Conger R, Siegler IC, Zhang X, Boardman JD, Frajzyngier Z, Stallings MC, Donnellan MB, Halpern CT, Harris KM [2015]. Population frequencies of the triallelic 5HTTLPR in six ethnically diverse samples from North America, Southeast Asia, and Africa. *Behav Genet*: 45[2]: 255–261. 10.1007/s10519-014-9703-5 25564228 PMC4348250

[37] Thomas D. [2010] Gene-environment-wide association studies: Emerging approaches. *Nature Rev Genet* 11: 259–272. 10.1038/nrg2764 20212493 PMC2891422

[38] Thapar A, Harold G, Rice F, Langley K, O’Donovan M. [2007] The contribution of gene-environment interaction to psychopathology. *Dev Psychopath* 39: 989–1004. 10.1017/S0954579407000491 17931430

[39] Moffitt TE, Caspi A, Rutter M. [2006] Measured gene-environment interaction in psychopathology. Concepts, research strategies, and implications for research, intervention, and public understanding of genetics. *Psychol Sci* 1: 5–27. 10.1111/j.1745-6916.2006.00002.x 26151183

[40] Monroe SM, Reid MW. [2008] Gene-environment interactions in depression research. *Psychol Sci* 19: 947–956. 10.1111/j.1467-9280.2008.02181.x 19000200

[41] Kessler RC. [1997] The effects of stressful life events on depression. *An Rev Psychol* 48: 191–214. 10.1146/annurev.psych.48.1.191 9046559

[42] Turecki G, Ernst C, Jollant F, Labonte B, Mechawar N. [2012] The neurodevelopmental origins of suicidal behavior. *Trends in Neurosc* 35: 14–23. 10.1016/j.tins.2011.11.008 22177979

[43] Brummett BH, Muller CL, Collins AL, Boyle SH, Kuhn CM, Seigler IC, Williams RB, Ashley-Koch A. [2008] 5HTTLPR and gender moderate changes in negative affect responses to tryptophan infusion. *Behav Genet* 38: 476–483. 10.1007/s10519-008-9219-y 18661222 PMC2689944

[44] Lin PY, Tsai G. [2004] Association between serotonin transporter gene promoter polymorphism and suicide: Results of a meta-analysis. *Biol Psychiatry* 55: 1023–1030. 15121487 10.1016/j.biopsych.2004.02.006

[45] Anguelova M, Benkelfat C, Turecki G. [2003] A systematic review of asociaiton studies investigating genes coding for serotonin receptors and the serotonin transporter II: Suicidal behavior. *Mol Psychiatry* 8: 646–653. 12874600 10.1038/sj.mp.4001336

[46] Brown GW, Harris TO. [2008] Depression and the serotonin transporter 5-HTTLPR polymorphism: A review and a hypothesis concerning gene-environment interaction. *J Affect Disorders* 111: 1–12. 10.1016/j.jad.2008.04.009 18534686

[47] Uher R, McGuffin P. [2010] The moderation by the serotonin transporter gene of environmental adversity in the etiology of depression: 2009 update. *Mol Psychiatry* 15: 18–22. 10.1038/mp.2009.123 20029411

[48] McGee RA, Wolfe DA, Yen SA, Wilson SK, Carnochan J. [1995] The measurement of maltreatment: a comparison of approaches. *Child Ab Neg* 19: 233–249. 10.1016/0145-2134(94)00119-f 7780784

[49] Henry B, Moffitt TE, Caspi A, Langley J, Silva PA. [1994] On the “remembrance of things past”: a longitudinal evaluation of the retrospective method. *Psychol Assess* 6: 92–101

[50] Williams DR, Gonzalez HM, Neighbors H, Nesse R, Abelson JM, Sweetman J et al [2007] Prevalence and distribution of major depressive disorder in African Americans, Caribbean Blacks, and Non-Hispanic Whites. *Arch Gen Psychiatry* 64: 305–315. 17339519 10.1001/archpsyc.64.3.305

[51] Hu XZ, Lipsky RH, Zhu G, Akhtar LA, Taubman J, Greenberg BD, et al [2006] Serotonin transporter promoter gain-of-function genotypes are linked to obsessive-compulsive disorder. *Amer J Hum Genet* 78: 815–826. 16642437 10.1086/503850 PMC1474042

[52] Murphy ML, Moya PR. [2011] Human serotonin transporter gene (SLC6A4) variants: their contributions to understanding pharmacogenomics and other functional GxG and GxE differences in health and disease. *Curr Opin Pharmacol* 11: 3–10. 21439906 10.1016/j.coph.2011.02.008 PMC3487694

