# Supplementary material for: Depression, Stressful Life Events, and the Impact of Variation in the Serotonin Transporter: Findings from the National Longitudinal Study of Adolescent to Adult Health (Add Health)
Source: PLoS One. 2016 Mar 3;11(3):e0148373. doi: 10.1371/journal.pone.0148373 (PMC4777542; doi:10.1371/journal.pone.0148373)
Supplement: S1 Table — (DOCX) [file pone.0148373.s001.docx]

| **S1 Table.** Domains and Items for Stressful Life Events (SLE) Measure. | |
| --- | --- |
| **Caspi et al (2003) SLE Items** | **Haberstick et al. SLE Items** |
| **Employment** |  |
|  |  |
| Long-term Unemployment | Received unemployment insurance as a source of income |
| Made redundant (laid off) |  |
| Lost job/company moved | Number of years worked uninterrupted – have been employed in each of the past two years; have been paid for work done in an employment situation. |
| Fired |  |
|  |  |
| **Financial** |  |
|  |  |
| Problems with debt | (In past 12 months) did not pay student loans or educational loans.  (In past 12 months) have any credit card debt? |
| Lack of $ to pay for food or household | (In past 12 months) did not pay full amount of gas, electricity, or oil bill due to not having enough money; did not pay mortgage or rent due to not having enough money; were without phone service. |
| Lack of $ for medical expenses | (In past 12 months) didn’t go see a doctor when needed to due to not being able to afford it; thought needed healthcare but didn’t get it because couldn’t afford it. |
| Difficulty paying bills | (In past 12 months) gas, electricity, or oil service was turned off because payments were not made. |
|  |  |
| **Housing** |  |
|  |  |
| Homelessness | (In past 12 months) ever evicted; ever stay at a homeless shelter; ever been homeless for a week or longer |
| Multiple residential changes | Number of address and state changes |
|  |  |
| **Health** |  |
|  |  |
| A disabling physical illness lasting a month or more | Number of hospitalizations in last year and past 5 years; hospitalization not for childbirth; number of seizures; diagnosis with cancer |
| A disabling injury | Number of violent injuries; disabling illness or limitation of activities which did not allow ability to work |
|  |  |
| **Relationships** |  |
|  |  |
| Involvement in a physically violent relationship | How often have you been hit; kicked, or slapped by you or your partner; have you or your partner sustained an injury because of a fight with your partner; frequency of times you or your partner insisted or made you have sexual relationship when you didn’t want to. |
| A break-up of a cohabitating, intimate relationship | Intimate relationship break up, divorce |
