# Supplementary material for: Depression, Stressful Life Events, and the Impact of Variation in the Serotonin Transporter: Findings from the National Longitudinal Study of Adolescent to Adult Health (Add Health)
Source: PLoS One. 2016 Mar 3;11(3):e0148373. doi: 10.1371/journal.pone.0148373 (PMC4777542; doi:10.1371/journal.pone.0148373)
Supplement: S2 Table — (DOCX) [file pone.0148373.s002.docx]

**S2 Tables**

| **S3A.** Sample Size and Frequencies for Independent and Dependent Variables – *Primary Analysis*. | | | | | | | | | | | | | | | | |
| --- | --- | --- | --- | --- | --- | --- | --- | --- | --- | --- | --- | --- | --- | --- | --- | --- |
|  |  | Depression | |  |  | Stressful Life Events | |  |  | Childhood  Maltreatment | |  |  | 5HTTLPR + rs25331 | |  |
| Level | N | | % | | N | | % | | N | | % | | N | | % | |
| 0 | 4096 | | 86.8 | | 1075 | | 22.8 | | 3637 | | 76.9 | | 1117 | | 23.6 | |
| 1 | 626 | | 13.2 | | 1560 | | 33.0 | | 794 | | 16.8 | | 2303 | | 48.8 | |
| 2 |  | |  | | 1117 | | 23.7 | | 293 | | 6.2 | | 1304 | | 27.6 | |
| 3 |  | |  | | 634 | | 13.4 | |  | |  | |  | |  | |
| 4 |  | |  | | 338 | | 7.2 | |  | |  | |  | |  | |

Note: Unweighted %.

| **S3B.** Sample Sizes for Independent and Dependent Variables – *Post Hoc Analyses.* | | | | | | | | | | | | | | | | |
| --- | --- | --- | --- | --- | --- | --- | --- | --- | --- | --- | --- | --- | --- | --- | --- | --- |
|  |  | Depression | |  |  | Stressful Life Events | |  |  | Childhood  Maltreatment | |  |  | Suicide Ideation | |  |
| Level | Males | | Females | | Males | | Females | | Males | | Females | | Males | | Females | |
| 0 | 2107 | | 1991 | | 595 | | 480 | | 1774 | | 1863 | | 2185 | | 2280 | |
| 1 | 205 | | 421 | | 813 | | 747 | | 407 | | 387 | | 127 | | 132 | |
| 2 |  | |  | | 537 | | 580 | | 131 | | 162 | |  | |  | |
| 3 |  | |  | | 263 | | 371 | |  | |  | |  | |  | |
| 4 |  | |  | | 104 | | 234 | |  | |  | |  | |  | |
