# Supplementary material for: Depression, Stressful Life Events, and the Impact of Variation in the Serotonin Transporter: Findings from the National Longitudinal Study of Adolescent to Adult Health (Add Health)
Source: PLoS One. 2016 Mar 3;11(3):e0148373. doi: 10.1371/journal.pone.0148373 (PMC4777542; doi:10.1371/journal.pone.0148373)
Supplement: S3 Table — (DOCX) [file pone.0148373.s003.docx]

**S3 Tables**

| **S4A.** Prevalence of depression as a function of 5HTTLPR genotype and number of stressful life events among Males (N = 2312). | | | | | | | | | | | | |
| --- | --- | --- | --- | --- | --- | --- | --- | --- | --- | --- | --- | --- |
| # of Stressful Life Events |  | L/L  5HTTLPR Genotype | | |  | S/L  5HTTLPR Genotype | | |  | S/S  5HTTLPR Genotype | | |
|  | N | | P | P ‡ | N | | P | P ‡ | N | | P | P ‡ |
| 0 | 143 | | 4.2 | 5.8 | 290 | | 5.2 | 3.6 | 162 | | 2.5 | 1.9 |
| 1 | 177 | | 8.5 | 13.3 | 422 | | 6.6 | 8.3 | 214 | | 6.5 | 5.1 |
| 2 | 134 | | 11.9 | 14.5 | 250 | | 8.8 | 9.3 | 153 | | 7.2 | 7.9 |
| 3 | 58 | | 15.5 | 19.8 | 134 | | 13.4 | 17.1 | 71 | | 19.7 | 28.9 |
| 4 | 24 | | 20.8 | 25.1 | 49 | | 30.6 | 36.6 | 31 | | 41.9 | 47.8 |
| Pr < † |  | | 0.019 | 0.255 |  | | 0.000 | 0.000 |  | | 0.000 | 0.000 |

‡ Weighted prevalence.

† Significance of the bivariate association between the number of stressful life events and depression.

| **S4B.** Prevalence of depression as a function of 5HTTLPR genotype and number of stressful life events among Females (N = 2412). | | | | | | | | | | | | |
| --- | --- | --- | --- | --- | --- | --- | --- | --- | --- | --- | --- | --- |
| # of Stressful Life Events |  | L/L  5HTTLPR Genotype | | |  | S/L  5HTTLPR Genotype | | |  | S/S  5HTTLPR Genotype | | |
|  | N | | P | P ‡ | N | | P | P ‡ | N | | P | P ‡ |
| 0 | 114 | | 9.7 | 14.7 | 220 | | 8.2 | 9.7 | 146 | | 8.2 | 13.2 |
| 1 | 175 | | 14.3 | 15.2 | 379 | | 9.5 | 9.1 | 193 | | 11.4 | 12.0 |
| 2 | 124 | | 15.3 | 19.9 | 292 | | 19.9 | 21.7 | 164 | | 17.1 | 15.8 |
| 3 | 105 | | 23.8 | 26.9 | 173 | | 30.1 | 29.2 | 93 | | 27.9 | 28.6 |
| 4 | 63 | | 36.5 | 34.1 | 94 | | 39.4 | 39.7 | 77 | | 37.7 | 43.0 |
| Pr < † |  | | 0.000 | 0.093 |  | | 0.000 | 0.000 |  | | 0.000 | 0.000 |

‡ Weighted prevalence.

† Significance of the bivariate association between the number of stressful life events and depression.
