# Supplementary material for: Depression, Stressful Life Events, and the Impact of Variation in the Serotonin Transporter: Findings from the National Longitudinal Study of Adolescent to Adult Health (Add Health)
Source: PLoS One. 2016 Mar 3;11(3):e0148373. doi: 10.1371/journal.pone.0148373 (PMC4777542; doi:10.1371/journal.pone.0148373)
Supplement: S4 Table — (DOCX) [file pone.0148373.s004.docx]

**S4 Tables**

| **S5A.** Prevalence of suicide ideation as a function of 5HTTLPR genotype and number of stressful life events among Males (N = 2312). | | | | | | | | | | | | |
| --- | --- | --- | --- | --- | --- | --- | --- | --- | --- | --- | --- | --- |
| # of Stressful Life Events |  | L/L  5HTTLPR Genotype | | |  | S/L  5HTTLPR Genotype | | |  | S/S  5HTTLPR Genotype | | |
|  | N | | P | P ‡ | N | | P | P ‡ | N | | P | P ‡ |
| 0 | 143 | | 2.1 | 1.8 | 290 | | 2.4 | 2.4 | 162 | | 1.9 | 1.7 |
| 1 | 177 | | 3.9 | 6.8 | 422 | | 4.3 | 5.7 | 214 | | 3.7 | 4.1 |
| 2 | 134 | | 5.9 | 9.9 | 250 | | 6.8 | 5.9 | 153 | | 7.2 | 6.9 |
| 3 | 58 | | 10.3 | 10.6 | 134 | | 11.9 | 11.3 | 71 | | 8.5 | 5.5 |
| 4 | 24 | | 29.2 | 37.5 | 49 | | 14.3 | 17.0 | 31 | | 9.7 | 11.4 |
| Pr < † |  | | 0.000 | 0.008 |  | | 0.000 | 0.036 |  | | 0.061 | 0.162 |

‡ Weighted prevalence.

† Significance of the bivariate association between the number of stressful life events and suicide ideation.

| **S5B.** Prevalence of suicide ideation as a function of 5HTTLPR genotype and number of stressful life events among Females (N = 2412). | | | | | | | | | | | | |
| --- | --- | --- | --- | --- | --- | --- | --- | --- | --- | --- | --- | --- |
| # of Stressful Life Events |  | L/L  5HTTLPR Genotype | | |  | S/L  5HTTLPR Genotype | | |  | S/S  5HTTLPR Genotype | | |
|  | N | | P-value | P-value ‡ | N | | P-value | P-value ‡ | N | | P-value | P-value ‡ |
| 0 | 114 | | 3.5 | 4.9 | 220 | | 1.4 | 2.3 | 146 | | 2.7 | 2.5 |
| 1 | 175 | | 3.4 | 3.2 | 379 | | 3.2 | 2.8 | 193 | | 4.2 | 3.1 |
| 2 | 124 | | 8.9 | 9.8 | 292 | | 3.4 | 3.5 | 164 | | 2.4 | 3.1 |
| 3 | 105 | | 9.5 | 6.1 | 173 | | 7.5 | 10.4 | 93 | | 7.5 | 7.3 |
| 4 | 63 | | 9.5 | 5.8 | 94 | | 24.5 | 26.7 | 77 | | 14.3 | 15.5 |
| Pr < † |  | | 0.084 | 0.093 |  | | 0.000 | 0.000 |  | | 0.001 | 0.005 |

‡ Weighted prevalence.

† Significance of the bivariate association between the number of stressful life events and suicide ideation.
