# Supplementary material for: Depression, Stressful Life Events, and the Impact of Variation in the Serotonin Transporter: Findings from the National Longitudinal Study of Adolescent to Adult Health (Add Health)
Source: PLoS One. 2016 Mar 3;11(3):e0148373. doi: 10.1371/journal.pone.0148373 (PMC4777542; doi:10.1371/journal.pone.0148373)
Supplement: S5 Table — (DOCX) [file pone.0148373.s005.docx]

S5 Tables.

| Regression Output for Depression Analysis – Full Sample. | | | | | | |
| --- | --- | --- | --- | --- | --- | --- |
|  |  |  |  |  |  |  |
|  | *b* | SE | *t* | *P* | 95% Confidence Interval | |
|  |  |  |  |  |  |  |
| Intercept | -3.3884 | 0.1936 | -17.50 | 0.000 | -3.7729 | -3.0039 |
| Sex (Female) | 0.5471 | 0.0989 | 5.53 | 0.000 | 0.3507 | 0.7435 |
| 5HTTLPR’ | 0.3975 | 0.0771 | 2.66 | 0.009 | 0.1010 | 0.6940 |
| SLE | 0.6744 | 0.0771 | 8.75 | 0.000 | 0.5214 | 0.8276 |
| 5HTTLPR’xSLE | -0.1607 | 0.0669 | -2.40 | 0.018 | -0.2936 | -0.0276 |
|  |  |  |  |  |  |  |

| Regression Output for Maltreatment Analysis – Full Sample. | | | | | | |
| --- | --- | --- | --- | --- | --- | --- |
|  |  |  |  |  |  |  |
|  | *b* | SE | *t* | *P* | 95% Confidence Interval | |
|  |  |  |  |  |  |  |
| Intercept | -2.3962 | 0.1403 | -17.08 | 0.000 | -2.6747 | -2.1177 |
| Sex (Female) | 0.7036 | 0.1069 | 6.58 | 0.000 | 0.4914 | 0.9158 |
| 5HTTLPR’ | 0.0803 | 0.0734 | 1.09 | 0.277 | -0.0657 | 0.2262 |
| Maltreatment | 0.4549 | 0.1421 | 3.20 | 0.002 | 0.1728 | 0.7372 |
| 5HTTLPR’x  Maltreatment | -0.0075 | 0.1141 | -0.07 | 0.948 | -0.2341 | 0.2191 |
|  |  |  |  |  |  |  |

| Regression Output for Suicide Ideation Analysis – Full Sample. | | | | | | |
| --- | --- | --- | --- | --- | --- | --- |
|  |  |  |  |  |  |  |
|  | *b* | SE | *t* | *P* | 95% Confidence Interval | |
|  |  |  |  |  |  |  |
| Intercept | -3.9248 | 0.3649 | -10.75 | 0.000 | -4.6495 | -3.2002 |
| Sex (Female) | -0.2723 | 0.1551 | -1.76 | 0.082 | -0.5803 | 0.0356 |
| 5HTTLPR’ | 0.3279 | 0.2276 | 1.44 | 0.153 | -0.1239 | 0.7798 |
| SLE | 0.5920 | 0.1245 | 4.76 | 0.000 | 0.3449 | 0.8392 |
| 5HTTLPR’xSLE | -0.0670 | 0.0900 | -0.74 | 0.458 | -0.2458 | 0.1117 |
|  |  |  |  |  |  |  |

| Regression Output for Depression Analysis – Males. | | | | | | |
| --- | --- | --- | --- | --- | --- | --- |
|  |  |  |  |  |  |  |
|  | *b* | SE | *t* | *P* | 95% Confidence Interval | |
|  |  |  |  |  |  |  |
| Intercept | -4.0648 | 0.3470 | -11.71 | 0.000 | -4.7543 | -3.3753 |
| 5HTTLPR’ | 0.8175 | 0.2674 | 3.06 | 0.003 | 0.2864 | 1.3487 |
| SLE | 0.9632 | 0.1451 | 6.64 | 0.000 | 0.6748 | 1.2515 |
| 5HTTLPR’xSLE | -0.3269 | 0.1178 | -2.78 | 0.007 | -0.5609 | -0.0929 |
|  |  |  |  |  |  |  |

| Regression Output for Maltreatment Analysis – Males. | | | | | | |
| --- | --- | --- | --- | --- | --- | --- |
|  |  |  |  |  |  |  |
|  | *b* | SE | *t* | *P* | 95% Confidence Interval | |
|  |  |  |  |  |  |  |
| Intercept | -2.4691 | 0.1967 | -12.55 | 0.000 | -2.8598 | -2.0783 |
| 5HTTLPR’ | 0.1952 | 0.1486 | 1.31 | 0.192 | -0.0999 | 0.9040 |
| Maltreatment | 0.4353 | 0.2359 | 1.85 | 0.068 | -0.0334 | 0.9040 |
| 5HTTLPR’x  Maltreatment | -0.0979 | 0.2193 | -0.45 | 0.656 | -0.5336 | 0.3378 |
|  |  |  |  |  |  |  |

| Regression Output for Suicide Ideation Analysis – Males. | | | | | | |
| --- | --- | --- | --- | --- | --- | --- |
|  |  |  |  |  |  |  |
|  | *b* | SE | *t* | *P* | 95% Confidence Interval | |
|  |  |  |  |  |  |  |
| Intercept | -3.5876 | 0.3302 | -10.87 | 0.000 | -4.2435 | -2.9318 |
| 5HTTLPR’ | 0.0453 | 0.2395 | 2.91 | 0.850 | -0.4305 | 0.5211 |
| Suicide Ideation | 0.3639 | 0.1252 | 2.91 | 0.005 | -0.0823 | 0.3486 |
| 5HTTLPR’x  Suicide Ideation | 0.1331 | 0.1084 | 1.23 | 0.223 | -0.0823 | 0.3486 |
|  |  |  |  |  |  |  |

| Regression Output for Depression Analysis – Females. | | | | | | |
| --- | --- | --- | --- | --- | --- | --- |
|  |  |  |  |  |  |  |
|  | *b* | SE | *t* | *P* | 95% Confidence Interval | |
|  |  |  |  |  |  |  |
| Intercept | -2.4666 | 0.2073 | -11.90 | 0.000 | -2.8789 | -2.0543 |
| 5HTTLPR’ | 0.1605 | 0.1817 | 0.88 | 0.380 | -0.2009 | 0.5219 |
| SLE | 0.5225 | 0.0830 | 6.29 | 0.000 | 0.3573 | 0.6877 |
| 5HTTLPR’xSLE | -0.0723 | 0.0775 | -0.93 | 0.353 | -0.2265 | 0.0818 |
|  |  |  |  |  |  |  |

| Regression Output for Maltreatment Analysis – Females. | | | | | | |
| --- | --- | --- | --- | --- | --- | --- |
|  |  |  |  |  |  |  |
|  | *b* | SE | *t* | *P* | 95% Confidence Interval | |
|  |  |  |  |  |  |  |
| Intercept | -1.6526 | 0.1346 | -12.27 | 0.000 | -1.9203 | -1.3848 |
| 5HTTLPR’ | 0.0137 | 0.0932 | 0.15 | 0.884 | -0.1718 | 0.1992 |
| Maltreatment | 0.4844 | 0.1518 | 3.19 | 0.002 | 0.1824 | 0.7863 |
| 5HTTLPR’x  Maltreatment | 0.0284 | 0.1308 | 0.22 | 0.829 | -0.2317 | 0.2886 |
|  |  |  |  |  |  |  |

| Regression Output for Suicide Ideation Analysis – Females. | | | | | | |
| --- | --- | --- | --- | --- | --- | --- |
|  |  |  |  |  |  |  |
|  | *b* | SE | *t* | *P* | 95% Confidence Interval | |
|  |  |  |  |  |  |  |
| Intercept | -4.5159 | 0.4987 | -9.05 | 0.000 | -5.5078 | -3.5241 |
| 5HTTLPR’ | 0.5775 | 0.3455 | 1.67 | 0.098 | -0.1097 | 1.2647 |
| Suicide Ideation | 0.7558 | 0.1746 | 4.33 | 0.000 | 0.4085 | 1.1031 |
| 5HTTLPR’x  Suicide Ideation | -0.2062 | 0.1237 | -1.67 | 0.099 | -0.4521 | 0.0397 |
|  |  |  |  |  |  |  |
