# Supplementary material for: Depression, Stressful Life Events, and the Impact of Variation in the Serotonin Transporter: Findings from the National Longitudinal Study of Adolescent to Adult Health (Add Health)
Source: PLoS One. 2016 Mar 3;11(3):e0148373. doi: 10.1371/journal.pone.0148373 (PMC4777542; doi:10.1371/journal.pone.0148373)
Supplement: S6 Table — (DOCX) [file pone.0148373.s006.docx]

**S6 Tables**

| **Table S7A.** Prevalence of depression as a function of 5HTTLPR genotype and number of childhood maltreatment events among Males (N = 2312). | | | | | | | | | | | | |  |
| --- | --- | --- | --- | --- | --- | --- | --- | --- | --- | --- | --- | --- | --- |
| # of maltreatment events |  | L/L  5HTTLPR Genotype | | |  | S/L  5HTTLPR Genotype | | |  | S/S  5HTTLPR Genotype | | | |
|  | N | | P | P ‡ | N | | P | P ‡ | N | | P | P ‡ |  |
| 0 | 417 | | 9.4 | 11.9 | 874 | | 7.2 | 8.1 | 483 | | 7.7 | 9.1 |  |
| 1 | 94 | | 9.6 | 16.8 | 209 | | 13.4 | 14.9 | 104 | | 9.6 | 7.9 |  |
| 2+ | 25 | | 12.0 | 14.0 | 62 | | 11.3 | 11.1 | 44 | | 20.5 | 21.8 |  |
| Pr < † |  | | 0.908 | 0.673 |  | | 0.012 | 0.066 |  | | 0.016 | 0.064 |  |

‡ Weighted prevalence.

† Significance of the bivariate association between the number of stressful life events and childhood maltreatment.

| **Table S7B.** Prevalence of childhood maltreatment as a function of 5HTTLPR genotype and number of childhood maltreatment events among Females (N = 2412). | | | | | | | | | | | | |  |
| --- | --- | --- | --- | --- | --- | --- | --- | --- | --- | --- | --- | --- | --- |
| # of maltreatment events |  | L/L  5HTTLPR Genotype | | |  | S/L  5HTTLPR Genotype | | |  | S/S  5HTTLPR Genotype | | | |
|  | N | | P-value | P-value ‡ | N | | P-value | P-value ‡ | N | | P-value | P-value ‡ |  |
| 0 | 445 | | 13.9 | 16.8 | 913 | | 15.9 | 15.7 | 505 | | 15.3 | 15.9 |  |
| 1 | 87 | | 26.4 | 26.2 | 186 | | 20.9 | 25.4 | 114 | | 23.7 | 28.3 |  |
| 2+ | 49 | | 36.7 | 38.9 | 59 | | 28.8 | 28.9 | 54 | | 24.1 | 30.5 |  |
| Pr < † |  | | 0.000 | 0.014 |  | | 0.014 | 0.018 |  | | 0.041 | 0.006 |  |

‡ Weighted prevalence.

† Significance of the bivariate association between the number of stressful life events and childhood maltreatment.
