# Supplementary material for: Depression, Stressful Life Events, and the Impact of Variation in the Serotonin Transporter: Findings from the National Longitudinal Study of Adolescent to Adult Health (Add Health)
Source: PLoS One. 2016 Mar 3;11(3):e0148373. doi: 10.1371/journal.pone.0148373 (PMC4777542; doi:10.1371/journal.pone.0148373)
Supplement: S1 Text — (DOCX) [file pone.0148373.s007.docx]

**S1 Text**

Genotyping information

Allele or repeat sizes for the 5HTTLPR ranged from 14R to 22R, with the most common being 14 or “short” and 16 or “long” repeat units. The 18R, 19R, 20R, and 22R are considered “extra-long” alleles and were binned with the long (16R) alleles. 5HTTLPR genotypes were coded as 0, 1, and 2 with higher numbers representing a greater number of long alleles.

For snp rs25331 the resulting functional variants of the L allele have been classified as L_a_ and L_g_ [1] and the 5HTTLPR tri-allelic locus with alleles S, L_a_, and L_g_. Allele frequencies were 42.2%, 47.6% and 10.2%, respectively. Following a previous report (2) we combined the resulting six two-locus genotypes into five two-locus genotypes: SS, SL_g_, SL_a_, L_g_L_a_, and L_a_L_a_. The genotype frequencies for these five genotypes were: 20.9%, 7.7%, 38.3%, 10.0% and 23.2%, respectively. The L_g_L_g_ genotype frequency was 1.3% and was combined with the SS genotype (1, 3). For the purpose of our analyses we created a 5HTTLPR genotype with three levels: (1) *SS + SL_g_* (referred to as s/s genotype), (2) *Sl_a_ + L_a_L_g_* (s/l genotype), and (3) *L_a_L_a_* (l/l genotype).

Polymerase chain reactions (PCR) contained two µl of DNA (20 ng or less), 1x Buffer II (ABI, Applied Biosystems, Foster City, CA), 1.8 mM MgCl_2_, 180 µM each deoxynucleotide (dNTP, NEB), with 7'-deaza-2'-deoxyGTP (deaza-GTP, Roche Applied Science, Indianapolis, IN) substituted for one-half of the dGTP, forward (fluorescently labeled) and reverse primers (concentrations in Table 1) and one unit of AmpliTaq Gold® polymerase (ABI) in a total volume of 20 µl. Forward and reverse primer sequences were: NED-ATG CCA GCA CCT AAC CCC TAA TGT (concentration: 600 nM) and GGA CCG CAA GGT GGG CGG GA (concentraten: 600 nM), respectively. Amplifications were performed using a modified (4) touchdown PCR method (5). A 95°C incubation for 10 min was followed by two cycles of 95°C for 30 s, 65°C for 30 s, and 72°C for 60 s. The annealing temperature was decreased every two cycles from 65°C to 57°C in 2°C increments (10 cycles total), followed by 30 cycles of 95°C for 30 s, 55°C for 30 s, and 72°C for 60 s and a final 30-min incubation at 72°C and a hold at 4°C.

**Supplement Literature Cited**

1. Hu XZ, Lipsky RH, Zhu G, Akhtar LA, Taubman J, Greenberg BD, *et al*. Serotonin transporter promoter gain-of-function genotypes are linked to obsessive-compulsive disorder. *Amer J Hum Genet* 2006; 78: 815-826.

2. Coventry WL, James MR, Eaves LJ, Gordon SD, Gillespie NA, Ryan L, Heath AC, *et al*. Do 5HTTLPR and stress interact in risk for depression and suicidality? Item response analyses of a large sample. *Am J Med Genet Part B.* 2009; 153B: 757-765.

3. Wendlend JR, Martin BJ, Kruse MR, Lesch KP, Murphy DL. Simultaneous genotyping of four functional loci of human SLC6A4, with reappraisal of 5-HTTLPR and rs25531. *Mol Psychiatry* 2006; 11: 224-226

**4.** Anchordoquy HC, McGeary C, Liu L, Krauter KS, Smolen A (2003) Genotyping of three candidate genes after whole-genome preamplification of DNA collected from buccal cells. Behav Genet 33: 73-78.

**5.** Don RH, Cox RT, Wainwright BJ, Baker K, Mattick JS (1992) “Touchdown” PCR to circumvent spurious priming during gene amplification. Nucleic Acids Res. 19: 4008.
